# Medication literacy and its social contextuality

**DOI:** 10.1177/13634593231211520

**Published:** 2023-12-05

**Authors:** Noémia Lopes, Carla Rodrigues, Elsa Pegado

**Affiliations:** Iscte - Instituto Universitário de Lisboa, CIES-Iscte, Portugal; IUEM – Instituto Universitário Egas Moniz, Portugal; Iscte - Instituto Universitário de Lisboa, CIES-Iscte, Portugal; University of Amsterdam, The Netherlands; Iscte - Instituto Universitário de Lisboa, CIES-Iscte, Portugal

**Keywords:** dietary supplements, medication literacy, pharmaceuticalisation, pharmaceuticals, social contextuality

## Abstract

This article aims to contribute to the discussion about medication literacy, by focussing on the social contextuality of the information mobilised in the use of medicines. We aim to explore the social construction processes of medication literacy, as an essential dimension for a more layperson-centred approach in the promotion of literacy in this field. This approach is justified by the growing social and cultural dissemination of medication use, the diversification of its uses beyond health and illness, and the increasing degree of lay autonomy in managing its use. The article is organised in two main sections. In the first section, we review the *social history* of medication literacy, including a discussion of the social contextuality of literacy phenomena. In the second section, the analysis of social contextuality is operationalised with a focus on information, covering: (i) ways of relating to institutional information and sources of information about medication; (ii) contexts of sociability in which information is shared and validated. This analysis is empirically supported by selected results from two research projects, conducted in Portugal, on the consumption of medicines and dietary supplements for performance purposes – that is, for the management and/or improvement of cognitive, bodily or relational performance.

## Introduction

The notion of medication literacy is a relatively recent category whose content refers to medicine use skills (Raynor, 2009). Although inseparable from health literacy, the emergence of this new category, and the proliferation of studies in this field (Cordina et al., 2018; Pantuzza et al., 2021; Pouliot et al., 2018; Raynor, 2009), signals a change in the social space of medication in everyday life.

The theme of medication literacy emerges in a context of an increasing intensification of the use of medication and an expansion of its applications beyond health and illness. In this context, known as the pharmaceuticalisation of everyday life (Williams et al., 2008), medication literacy arises as a relevant, analytically autonomous topic, distinct from its origins in health literacy. The challenges presented by this new framework imply the problematisation of the concept of medication literacy itself, particularly in relation to its scope and its limited ability to capture the competencies it aims to assess.

Subject to a metric and functional conception of competencies, medication literacy has been a tool of limited reach for understanding the multiple dimensions of its object of study. Among these dimensions, the social contextuality of literacy practices has been relatively underexplored. Therefore, broadening the focus to include social contextuality becomes crucial for understanding the processes of production and mobilisation of medication literacy.

This article aims to contribute to the discussion about medication literacy, by emphasising the social contextuality of the information mobilised in medicine use. We aim to explore the social construction processes of medication literacy, as an essential dimension for a more layperson-centred approach in the promotion of literacy in this field. The article is organised in two main sections. In the first section, we review the *social history* of medication literacy, including a discussion of the social contextuality of literacy phenomena. In the second section, the analysis of social contextuality is operationalised with a focus on information, covering: (i) ways of relating to institutional information and information sources about medicines; (ii) sociability contexts in which information is shared and validated. This analysis is empirically supported by selected results from two research projects, conducted in Portugal, on the use of medication and supplements for performance purposes – that is, for the management and/or improvement of cognitive, body or relational performance.

## Medication literacy: From functionality to contextuality

Over the past two decades, the surge in studies on literacy within the field of health and, more recently, the field of medication, has broadened the problematisation of the conceptual and analytical scope of the notion of literacy in both health and medication. Such increased problematisation mirrors the transformations that have occurred in the field of health in general, and in medication in particular. In both cases, these changes confer a new centrality to information in the professional-patient relationship.

Early formulations of medication literacy focussed on the acquisition of skills in the use of information about medicines, particularly the use of medication leaflets (Raynor, 2009; Raynor et al., 2007; Sauceda et al., 2012). The limited scope of this conception of literacy, as pointed out by different authors, lies in its strictly functional nature and in the individualisation of competences, which emphasises the individualised transmission of information (Cordina et al., 2018). This conception atomises the subject from its social contextuality, thereby erasing the effect of such contextuality on the conditions of literacy construction and reducing it to an individual attainment of cognitive skills (Samerski, 2019). Within this conception, individuals are seen as passive recipients expected to comply with expert guidance. This excludes the possibility of capturing the wider range of informational and experiential resources that individuals mobilise in their decisions regarding health and medication.

Functional conceptions of literacy started to be questioned following the emergence of ‘New Literacy Studies’ (Collins, 2000; Gee, 2015), through which the social contextualisation of literacy practices was brought to the debate. The notion of literacy and its angle of analysis became wider: ‘(. . .) literacy should be studied in an integrated way in its full range of contexts and practices, not just cognitive, but social, cultural, historical, and institutional, as well’ (Gee, 2015: 35). The notion of social context, however, comes to figure only as a referential notion, confined to a mediating function in the ways of accessing information, without considering its effect on structuring the forms of assimilation and application of said information. In medication literacy, this emphasis on the social space of an individual’s ‘decision-making’ shifted the notion away from a functional view of compliance – acting in strict conformity with expert guidelines – and brought it closer to the idea of ‘informed decision-making’. Following this approach, medication literacy has more recently been defined as: ‘the degree to which individuals can obtain, comprehend, communicate, calculate and process patient-specific information about their medications to make informed medication and health decisions in order to safely and effectively use their medications (. . .)’ (Pouliot et al., 2018: 797). However, despite coming close to a social contextualisation conception of literacy, the emphasis on informed decision-making operates only a minimalistic notion of social context. As Chinn (2011: 61) points out, this interactive nature of literacy still remains conceptualised as ‘(. . .) the interaction between individuals and information’. Thus, a structurally individualised and cognitive conception of literacy prevails.

Some of the most effective contributions to the framing of social context as a part of ‘literacy events’ (Papen, 2009) – understood as ‘socially situated events’ (*idem*) – come from so-called ‘Critical Health Literacy’ (Chinn, 2011; Nutbeam, 2008). In this field, unlike previous approaches, health literacy is seen as a dimension mobilised beyond a strict medical context and health organisations. The importance of broadening the focus of health literacy to go beyond health spaces and the strict relationship with professionals is underlined by Chinn (2011: 60), when she states that the realisation of health ‘. . .is the sum of many everyday judgements and activities outside the hospital or doctor’s consulting room’. The ubiquity of health literacy is also highlighted by other authors: ‘(. . .) health literacy includes information and decision-making skills occurring in the workplace, in the supermarket, in social and recreational settings (. . .)’ (Peerson and Saunders, 2009: 289). Despite the broadening of the concept of literacy in this approach, the processes and effects of the social context in the production of health literacy continue to be overlooked.

### Information versus knowledge

One of the critical points regarding how both medication literacy and health literacy models are conceptualised lies in the overvaluation of the provision and accessibility of information, without considering the sociocultural processes involved in the ways of assimilating and mobilising this information. As Samerski (2019) states, ‘information seeking and health related actions are strongly determined by concrete situations (. . .) and by interpersonal relationships with informal or professional helpers’.

Redirecting the focus from information to the knowledge mobilised in everyday (health) life is also highlighted by Samerski (2019) as a necessary analytical investment that remains underexplored. Health knowledge, as an analytical dimension in literacy studies, thus assumes new visibility and relevance. Besides being one of the elements for capturing the social contextuality of health and/or medication practices, it also allows for a more insightful and consistent analysis of the information validation and operationalisation processes generated in daily social practices.

Considering the production of health and/or medication literacy based on how everyday knowledge is structured brings two reference systems into play: the *lay referential system* (Freidson, 1984), associated with information and knowledge assimilated in the shared experiences taking place within the sociocultural contexts of everyday life, and the *expert systems* (Giddens, 1992), associated with professional and scientific knowledge. The interchangeability of information emanating from each of these systems is found in different modalities of health and medicine knowledge in everyday actions; that is, there is a practical knowledge inherent to the socially and culturally shared experience of corporeality which remains as a resource for action, despite the search for and assimilation of expert information.

Sociological research in this domain has supported the centrality of the bodily experience as a dimension of lay health and/or medication knowledge, using different designations: somatic knowledge; ontological knowledge; practical knowledge (Baszanger, 1998; Lopes, 2009; Samerski, 2019). Bodily responses constitute the locus of practical control and validation of the efficacy of the medication used (Lopes, 2009); institutional information is filtered and re-evaluated through what Baszanger (1998) calls *practical lay control*. To this modality we can add other composite forms of knowledge where expert information and somatic experience combine and reconfigure themselves into forms of literacy. In a study on self-medication practices (Lopes, 2009) it was possible to identify forms of appropriation and assimilation of expert knowledge – medical and pharmaceutical information – that configure cognitive constructions based on a double affiliation: they not only result from a reflexive appropriation of expert references, but also from the reassessment of the effectiveness of those references within the framework of practical experiences. This composite nature of cognitive constructions in the relationship with medication has also been observed in more recent studies (Rodrigues, 2016, 2020).

The focus on knowledges allows us to highlight the dimension of autonomy that persists in individuals’ relationships with health and medication. This autonomy is generated by individuals’ sociocultural contexts, by their own experience of corporality and by the somatic knowledge emanating from it. As Baszanger (1998) states, this is an autonomy materialised in the permanence of a lay perspective, which is present in the interaction with the expert system. In this sense, the instrumental perspective of individuals’ empowerment underlying the conception of literacy – centred on information but decontextualised from the social processes mediating the assimilation and reconversion of information in daily practices – appears to have limited reach and effectiveness.

Considering the social construction of lay knowledge as a structuring vector of literacy practices is a key exercise for the formulation of more effective and more layperson-centred literacy policies in health and medication.

## Medication in daily life: Beyond health and illness

The relevance attributed to medication literacy is inseparable from the growing dissemination of medication in everyday life. This growth in the availability and use of medication has given rise to new forms of lay autonomy in the management and use of medicines, and has bestowed particular centrality to information and informed decision-making (Bissell et al., 2000; Cordina et al., 2018). In this framework, medication literacy has acquired a new social instrumentality.

The progressive deregulation of access to several categories of medicine, as ‘over-the-counter’ medicines, beginning in the 1980s in most European countries (Barber, 1993; Bissell et al., 2000; WHO, 1988), has helped to reconfigure individuals’ relationship with medicines and with professional mediation. Individual responsibility for decisions about medicine use signals an institutional attribution of a margin for lay autonomy, supported and legitimised by new duties relating to information transmission by professionals, and information acquisition by laypeople.

Despite the increase in over-the-counter consumption, this has been a relatively marginal domain in literacy studies. Most studies have focussed on prescribed medication, with little research on lay knowledge and conceptions of over-the-counter medicines (Bissell et al., 2001). Yet, this changing access to medication calls for a broader scope of studies on medication literacy, and for overcoming the ethno-professional (Lopes, 2009), or strictly functional, conception that prevails in available studies. This requires considering the increasingly diverse social uses of medication, and the expert and lay informational and experiential sources of knowledge mobilised in medication choices.

The increasing use of medication beyond health and illness is yet another factor that justifies the need to problematise medication literacy. These medication practices involve the so-called ‘lifestyle drugs’ (Fox et al., 2009), or ‘performance consumptions’ (Lopes et al., 2015, 2017; Pegado et al., 2018), predominantly associated with well-being and (cognitive, bodily, relational) performance improvement goals. The cultural dissemination and diversification of medicine use and its purposes has received significant theoretical attention, as part of pharmaceuticalisation processes (Fox et al., 2009; Rodrigues et al., 2019; Lopes et al., 2015; Williams et al., 2008). The increasing pharmaceuticalisation of everyday life not only helps expand the space for lay autonomy and individual responsibility for medication choices, but also contributes to the multiplicity of available information sources.

These new performance consumptions highlight the importance of considering the informational and cognitive references mobilised by individuals in literacy assessment models. It is, therefore, important to consider the limitations of conventional literacy studies which, as Peerson and Saunders (2009: 287) point out, have nothing to say about non-ill individuals and do not consider the ‘health-related’ or medication-related decision-making strategies individuals use in the pursuit of ‘keeping well’.

Finally, natural medicines and dietary supplements are another growing segment of consumption that have been omitted or residual in studies on medication literacy. These types of consumption assume particular expression in performance management or improvement, and are generally used alternatively or complementarily with pharmaceuticals (Rodrigues et al., 2019; Lopes, 2010; Lopes et al., 2015). The transfer of information and knowledge between the natural and the pharmacological fields appears as a new social framework that justifies further study in medication literacy.

Having explored the factors that support the relevance of problematising the notion of medication literacy, and situating it in its social contextuality, we now turn to presenting empirical research results related to two dimensions of literacy construction. One dimension refers to the institutional sources of information about medication used in daily life, with a focus on the use and value assigned to the leaflets’ information. The other dimension concerns contexts of sociability, analysed as spaces for sharing information and experiences about medication, where a conjunctural or transitory validation of information takes place.

The empirical support for this approach is based on selected results of two studies (hereafter referred to as study l and study 2), which included a component on information sources regarding the use of medicines and supplements in health and/or performance consumption. Study 1 explores the use and value assigned to the information provided in the leaflets of medicines and supplements, based on data collected through a questionnaire. Study 2 delves into the plurality of informal information sources that shape practical knowledge about medicines and supplements, shared in contexts of sociability, drawing upon data collected from interviews. These findings illuminate under-explored social dimensions of medication literacy, namely the intersection of various sources and the margins of lay autonomy in the use of information.

## Study l – On medication leaflets: Uses and social attributed relevance

Despite the plurality of sources of information on health and medicines, research shows that individuals value the available sources differently, and mobilise them in diverse ways when making consumption choices. Institutional sources, whether resulting from direct interactions with doctors or pharmacists, or in their mediated form, such as medication information leaflets, rank high in the hierarchy of importance that individuals assign to them as sources of knowledge (Clamote, 2010, 2015).

Sociological literature on perceptions and uses of information available in leaflets or on medication packaging is relatively scarce, except for rare analytical incursions with a more critical perspective on classical approaches (Dixon-Woods, 2001). In turn, studies on the readability of leaflets abound (Pires et al., 2015). Adopting a medical or pharmaceutical perspective, several of these studies draw attention to the poor readability of leaflets and the need for linguistic improvements (Herber et al., 2014; Pires et al., 2015), emphasising readability as an essential component for a rational and safe use.

The indicators that underpin the following analysis come from a project on medicines and supplements use in high-pressure professions^1^. The data presented are from one section – focussed on medicine and supplement leaflets or packages– of a larger questionnaire that also included sections about work pressure factors and the use of medicines and supplements for performance purposes. The anonymous online questionnaire was applied to a sample of individuals in the labour market in the urban region of Lisbon and Oporto (*n* = 340). It was sent via professional associations between January and December 2020. Written informed consent was obtained from all participants in the study. The survey sample comprises 72.6% men and 27.4% women. Concerning their age, 26.5% were under 40, 40.9% were 40–49, and 32.6% were 50 or older. In terms of school level, 55.9% had secondary education or less, while 44.1% completed university studies.

In this section, the analysis is focussed on respondents’ use and perceptions of information leaflets for both prescribed and non-prescribed medicines, as well as dietary supplements. That is, the information detailing patient/consumer directions for proper use of medicines/supplements, including their approved/alleged uses, available formulations and dosages, administration dose and schedule, contraindications, and potential adverse reactions^2^.

The analysis is structured around three dimensions. The first two correspond to the uses of information in medication leaflets and the perceptions of this information. Both are conceived as forms of knowledge construction about medicines through proactive individual searches. The third dimension involves comparing individuals’ relationship with information about pharmaceuticals and supplements, a line of analysis that remains underexplored in medication literacy studies.

### The uses of the information in medication leaflets: Reading and attributed utility

The regularity with which the information in medication leaflets or packages is read indirectly measures the interest in this information. Table 1 shows that this frequency was quite high (more than 70% including ‘several/many times’ and ‘always’), for both prescribed and non-prescribed medicines. A prescription does not, therefore, invalidate the reading of the information, possibly through a logic of confirmation and/or complementarity.

**Table 1.** Regularity of reading the leaflets of the medicines taken.

	Prescribed by a doctor (%)	Not prescribed by a doctor (%)
Never	5.2	7.2
Very rarely/rarely	21.1	17.6
Several/many times	32.4	32.8
Always	41.3	42.4
Total^a^	100.0 (327)	100.0 (290)

a Differences in totals are due to the number of non-responses.

The reading rates above are consistent with the results of previous research in Portugal (Clamote, 2010), as well as in other countries such as the UK (Raynor et al., 2007) and the USA (Nathan et al., 2007).

The various types of information in medication leaflets were mostly perceived as useful (Table 2). Except for ‘composition’, all other sections appeared as ‘always useful’ in 55% to 65% of the cases. It would be important to qualitatively explore in future studies the meanings attributed to this usefulness and how they shape consumption practices.

**Table 2.** Usefulness of the information in medicines leaflets.

	Not at all/Rarely/Seldom useful (%)	Often/Almost always useful (%)	Always useful (%)	Total (%)^a^
Purposes	7.2	34.9	57.9	100.0 (321)
Composition	28.4	32.7	38.9	100.0 (306)
How to use	5.0	29.5	65.5	100.0 (322)
Secondary effects	9.4	30.4	60.2	100.0 (319)
Interactions with food or other medication	11.6	33.3	55.0	100.0 (318)

a Differences in totals are due to respondents who indicated they had no opinion on each item.

Regardless of this apparent consensus, there were noteworthy differences between the various types of information. First, slightly higher value was given to information on ‘how to use’, favouring a type of instrumental information with immediate application in consumption practices. Second, a lower rating was given for more technical information, namely ‘composition’, which is not directly *usable* when taking the medicine.

The importance of information on side effects and on interactions with other drugs or products has also been highlighted in other studies (Grime et al., 2007; Nathan et al., 2007). Indeed, this seems to be one of the main reasons why people consult medication leaflets (Clamote, 2015), reflecting the relatively high risk perceptions about pharmaceuticals (Raposo, 2010). Other research has shown reactions of fear and uncertainty caused by reading information about side effects; discussing how this can be presented with the aim of minimising possible perverse effects is therefore important (Herber et al., 2014).

### Perceptions of information on medication leaflets: Quality and trust

In European Union countries, medication leaflets need to be organised by sections with specific requirements and their wording must be simple and clear (European Comission, 2009). The leaflets are subjected to readability tests with small groups of users, preferably with lower levels of general literacy (Pires et al., 2015).

In the present study, the overall assessment of the quality of information on medication leaflets and packages was quite positive (see Table 3). Most attributes of information were evaluated relatively favourably. However, there was still a considerable percentage of respondents for whom the information was ‘unclear’ and ‘incomplete’. Moreover, about two-thirds considered the information ‘too technical’ (see also Grime et al., 2007). Studies on leaflet readability have shown that this aspect of information is one of the most sensitive, as it can compromise understanding of the written information on medicines. Underlying these studies is the idea that it would be enough to use a language accessible to patients for them to understand it, and to use the medicines appropriately. Therefore, the contextually situated agency of individuals, in the appropriation of this information and in the construction of knowledge about medicines, is omitted.

**Table 3.** Evaluation of the information in medicines leaflets.

	Disagrees (%)^a^	Agrees (%)^a^	Total (%)^b^
It is unclear	57.2	42.8	100.0 (306)
It is too technical	33.4	66.6	100.0 (311)
It is incomplete	62.9	37.1	100.0 (294)
It is clarifying	16.3	83.7	100.0 (312)
It is essential to know what is being used	5.7	94.3	100.0 (314)

a Original scale of six categories (Strongly disagree/Disagree/Partially disagree/Partially agree/Agree/Strongly agree), grouped into two.

b Differences in totals are due to respondents who indicated they had no opinion on each item.

Trust in the information in medication leaflets is quite high: about 75% of respondents declared that they have great or total confidence. However, the number of those who had reservations was not negligible. This shows there are still margins of uncertainty, whose origin needs to be studied.

### Medicines and dietary supplements: Similarities and contrasts

As mentioned above, the use and evaluation of information found in leaflets or packages of supplements was also included in this study. These products are not produced by the same chemical and pharmacological synthesis processes as pharmaceuticals, nor are they governed by the same rules that regulate their manufacture, labelling and marketing. Additionally, and although they may present beneficial effects to health, they cannot state prophylactic properties, nor claim the treatment or cure of diseases or symptoms^3^. These differences introduce, from the outset, an unequal institutional legitimation that does not always coincide with social legitimation. Two other criteria play a role in the construction of social legitimacy. First, the place of sale, that is, whether it is a pharmacy, parapharmacy, health food store or even a supermarket (Lopes, 2010). Second, whether there is a prescription, and the status of the prescriber or the adviser.

While the inclusion of a leaflet in medication packages is mandatory, for supplements there is no such requirement, even if in most cases they are accompanied by one. Thus, to ensure meaningful comparison of the uses and evaluations of information accompanying medications and supplements, the information contained in on packages was also considered.

The comparison between medicines and supplements reveals similarities in the uses of information – in terms of rates of reading and usefulness – but contrasts in the perceptions of that information – in terms of quality and trust. In fact, in terms of information reading frequency, there are no significant differences when compared to pharmaceuticals, that is, respondents indicated that they frequently read supplement leaflets or packages, whether or not recommended by a doctor or health professional (74.6% and 75.3%, respectively). In other words, the differences in the institutional status of supplements do not lead to devaluation of their accompanying information nor to their greater appreciation in the absence of an expert referral. The same applies to the usefulness of the various sections of information. As with pharmaceuticals, the item ‘how to use’ (93.2%) tops the ranking of usefulness, while the ‘composition’ (77.6%) was considered as relatively less useful.

In terms of the assessment of quality of information, supplements were evaluated less positively in almost all attributes under analysis. Noteworthy is the fact that information was considered ‘unclear’ (54%) and ‘incomplete’ (55.5%). However, in contrast with pharmaceuticals, 46.6% of respondents disagreed that supplement information is ‘too technical’.

Finally, the biggest contrast concerns the level of trust in the information, which was much lower for supplements than for pharmaceuticals. Only 40% of respondents said that they have a great or total trust in the information on supplement leaflets or packaging, compared to about 75% for medicines. This disparity is consistent with the perception that supplement leaflets or packages contain incomplete information, but it is also likely to be associated with the social credibility invested in them, which, not being validated by expert sources, requires other circuits for building trust.

Indeed, leaflets are of course only one of the informational sources that can be mobilised for the construction of knowledge about medicines. The sociological approach to medication literacy, as developed in this article, requires the consideration of a wider and more varied range of sources – expert, institutional, informal, and others – that are part of the social contextuality that shapes individuals’ relationships with medicines. In this framework, sociability networks, as developed in the following section, constitute an important context for information sharing and knowledge construction.

## Study 2 – The role of sociability networks in information sharing and knowledge production

In this section, we use qualitative data from a project on performance consumptions amongst young individuals^4^. Semi-structured interviews with 45 participants (aged 18−29 years) were conducted to analyse the ‘informational trajectories’ behind individuals’ consumption practices of medicines and supplements. We paid particular attention to contexts of sociability as privileged social spaces for information sharing and knowledge production. These young adults were either undergraduate students (46.5%) or young workers in call centres or in megastores without a university education (53.5%). These interviews were conducted in Portugal, between June 2013 and February 2014. They were all transcribed and a thematic analysis was undertaken. The interviews focussed on the use of (and dispositions for using) medicines and supplements for a variety of performance management purposes, and explored different contexts of use (and social legitimacies for using), access to and use of a range of information sources, and risk perceptions and management.

The contexts of sociability considered in this analysis relate to different spaces of action and of social interaction between individuals, in various spheres of their daily lives – at home, at work, at the gym, among others – where relationships of varying levels of social proximity are developed. It is within these social spaces that the circulation of information, ideas and points of reference take place and gain meaning. It is, therefore, important to understand the type of information and knowledge shared (and produced) within these networks, as well as to situate their role amongst the variety of other sources of information and recommendation, which may include healthcare professionals (such as medical doctors and pharmacists), shop attendants, information leaflets, and the internet, among others.

The relevance of the contexts of sociability in setting up, or in interpreting, the conditions for the potential need for performance consumptions, came out as key, not only in structuring forms of use but also in the process of knowledge construction around it. Various examples were found among study participants: teachers’ and colleagues’ recommendations to improve memory, concentration or sleeping patterns in universities and schools; personal trainers’ (PTs) and peers’ suggestions for body-related enhancement products in gymnasiums; and colleagues’ advice in workplaces to help deal with stress and other work-related issues. One of the examples came from a former member of the police force, who described how body strength and physical appearance had mattered, and how it had produced the need to resort to certain substances:

‘the guys there want to be more robust, bulkier, because the content of the missions entrusted to us is more for the frontal shock, the image, that deterrent shock through the image. So, powerful men are wanted, so to speak, and it’s a group behavior’. (M. 26 years, call centre)

In the case described above, the perceived pressure to meet the expectations of strong and robust bodies, resulted in an ‘almost mandatory’ use of different substances: from protein-based supplements to injectable hormone-based products. In such contexts, the circulation of information about what one needs, the best products, their effects and side effects, was abundant. In some cases, the sources of information were also the points of access to certain products, constituting, as Clamote (2015: 48) describes, ‘total contexts’^5^ in the organisation of consumptions practices: ‘inducing their need, referencing the resources to respond to it, and providing access to them’. While this does not mean that this informational circuit excludes other sources of information and knowledge – which often it does not – the sociability network established in such contexts worked as a prominent source of knowledge construction.

The above case is similar to what can be found in many gymnasiums, where the role of personal trainers as performance consumption advisers is more salient. This is so, not only in inducing use and providing guidance, but also in validating information collected through other sources:

‘I think it’s harmful to my health. (. . .) They’d told me about a powder to pour into milk, in a supplement to put on weight, but no. *[Interviewer: Who told you?]* The medical doctor told me about it, but then I informed myself at the gym [with the gym coach] about this situation and he told me that it’s not advisable’. (M. 22 years, megastore)

This example describes a situation where a coach’s advice, which went against a medical doctor’s recommendation for a supplement to gain muscular mass, prevailed. This is illustrative of how medical doctors’ authority in individuals’ decisions is but one among a ‘pluralism of expertise’ (Giddens, 1991), especially in certain performance consumptions (Monaghan, 1999; Clamote, 2015; Raposo and Rodrigues, 2021). Hence, the importance attributed to certain information sources varies according to the kind of consumption; in this case, both the technical and experiential knowledge of PTs in a gym can play a more prominent role in informing and (de)legitimising certain consumptions than that of medical doctors.

Family members also play a key role, not only in leading, but also in mediating the search for and in interpreting information related to performance consumptions (as well as therapeutic consumptions, cf. Clamote, 2010; Lopes, 2009; Rodrigues, 2016):

‘I only started taking Valdispert because [of] my [figure-skating] coach. I just imagined myself in the competition and I couldn’t sleep and was nervous. She said “*Look, buy Valdispert, take half an hour before, because it’s a natural product. . . It won’t make you sleepy, it will only calm you down so you can sleep”*. At the pharmacy, my mom immediately asked how it worked. I didn’t search [for more information], we went there to ask: “*So, what is this made of? How is it taken? Is it strong? Is it not?*” We read the little paper and we asked the pharmacist for information, of course. It wasn’t [just] because the coach told me. Of course, we did a pre-evaluation before we bought this’. (F. 20 years, engineering student)

The example above illustrates how knowledge is collectively constructed. It also shows some of the nuances around the role played by each of the sources and actors involved in such informational paths. While the coach’s recommendations are framed within the contextuality of the performance itself – and on whose expertise trainees often have to rely – more concrete information about the suggested substance was provided by trusted institutional sources, such as the pharmacist and the medicine leaflet. The mother also appears along the informational trajectory, helping evaluate all collected information. Indeed, mothers often have a key role as ‘lay advisors’ in medicine use, especially among youth or young adults. Such a role incorporates a level of trust which goes beyond the trust attributed to professionals’ expertise and technical knowledge, and relies more on other forms of social proximity and close relational ties (Rodrigues, 2016).

The strategies used to search for information sometimes also varied in relation to whether the substance was a pharmaceutical or perceived as a natural product. While the idea of a relative innocuity attributed to natural medicines or products (also found in other studies, e.g. Raposo, 2010, 2016; Rodrigues, 2016) was shared by many of the participants, it did not necessarily prevent them – as shown in the example above – from asking for advice or searching for information about their effects and side-effects. In some cases, however, the little (especially technical) information accompanying certain products required different strategies:

‘In the case of medicines, I don’t [use the internet]. Usually, the leaflet is enough for me. Therefore, I get the advice from the pharmacist and the leaflet. In the case of natural products, I can eventually do a little more research, as I don’t have access to the brochure. So, I can use the internet, knowing that it will never be very credible, but. . .I try to consult various sites and try to understand if they really match’. (M. 21 years, call centre)

While there was a preference to use institutional sources of information, such as consulting health professionals and reading the consumer information leaflets, the lack of such sources with many natural products led to more active internet searches. Even though the internet was not seen as a ‘very credible’ source of information, comparing the content found on different websites made it possible to build more consistent knowledge.

As analysed in the previous section, the information leaflets were a valuable source of information. Many of the study participants consulted them as one of the informational steps they would take to clarify and complement particular product features. These more standard and technical types of information found in leaflets are often complemented by other more valued and meaningful forms of knowledge for consumers. This includes information that is more specific to their own lifestyles and body conditions, but also information that is based on more ‘concrete’ forms of experiential knowledge (Brown and Calnan, 2012):

‘I always try to be accompanied by colleagues and friends who compete in high competition, also to find out how they evolved, how it started, what they took. And then I research about these types of products and see how they fit into my activity. I see on the internet, see the chemical compounds, the side effects they have. I also look for an answer at the level of each person, how they have been feeling. Much more than reading a simple package insert’. (M. 24 years, call centre)

Besides highlighting the role of particular contexts of action and interaction – in this case, the gym – in generating both consumption and knowledge, the example above is illustrative of how different forms of knowledge (more technical or experiential), provided by different sources of information, were selected and articulated to more quickly and safely achieve certain performative goals. It particularly highlights the importance of socially shared experiences as a main reference in performance consumptions, through both personal interactions and online blogs and discussion forums. In such online sources, study participants were mostly interested in reading about the popularity of particular products, and especially how others had experienced their effects and side-effects:

‘Of course, when I saw that [the protein-based supplement] was good for me, I informed myself, I looked it up on blogs, I was careful to know if it was natural, if it was a chemical, what the composition was. I looked in forums to see if that product was known to cause any side effects and, if so, what it provoked. Indications from other consumers who might eventually share with me a positive or negative experience’. (M. 26 years, call centre)

The internet was often used as a source with mixed levels of credibility. While information provided in certain institutional websites was mostly perceived as reliable, though sometimes too technical and difficult to understand, opinion forums and blogs were seen as useful and valuable for some, but unreliable for others. Hence, despite the general importance attributed to the experiential knowledge shared by other consumers, in many cases that was only meaningful if it was shared by someone they trusted within their personal network:

‘There are many websites that aren’t reliable. So, we have to go to the most reliable websites, because on these sites they actually say the components, what [it] is used for, what the side effects are, such as in the information leaflets. . .But even so, we always like to look for experience, I think it’s always better to talk to someone who really had the experience than really just reading anything on the internet. I really prefer to contact those I trust and have already tried’. (F. 21 years, pharmaceutical science student)

Along with the desirable combination of technical information and shared experiential knowledge, what this quote also highlights is the importance of selecting information from trustworthy sources, both institutional and interpersonal. In the case of information provided by those who used the products, a distinction is made between ‘opinions’ from those whose interests, subjectivities and modes of interpretation are unknown to the reader, and the ‘experience’ of those within an individual’s sociability network whom they trust. Such reliance is based on a more direct and less mediated face-to-face communication (Brown and Calnan, 2012) with whom this consumption may be ‘part of a wider set of shared practices’ (Rodrigues et al., 2019: 1015).

While such knowledge exchanges, often combined with other recommendations, are important points of reference for initiating (or adjusting) consumption, direct experiences of use and individuals’ own bodily responses (Lopes, 2009; Rodrigues, 2016; Samerski, 2019) are an additional source of knowledge:

‘I usually trust a little bit in what people say, otherwise I end up not buying things. But I always take my own conclusions. So, I take it, it gives me experience, and I see. . . I always start with the smallest dose of the product and I see how I am reacting. Then I increase up to the normal dosage’. (M. 24 years, call centre)

As the example above shows, while most performance as well as therapeutic consumptions are initiated based on a variety of sources and forms of knowledge, in many cases, it is through individuals’ own bodily responses that dosages are regulated and adjusted. In such cases, consumers’ own bodies become a ‘locus of experimentation’ (Rodrigues et al., 2019) and a more concrete way of assessing the (side-) effects of certain products. Hence, this analysis shows how individuals’ knowledge around medicines and supplements is constructed through different informational trajectories and within particular contexts of sociability.

## Final notes

As we have demonstrated in this article, the growing presence of medication in everyday life, both in the form of pharmaceuticals and supplements, justifies the growing relevance of medication literacy studies. In this context, there is a need to problematise the notion of medication literacy and expand it beyond the strict metric and functional conceptualisation that has governed it. Given that the construction of literacy indicators derives from the notion of literacy associated with them, we argue that the problematisation proposed in this article is a requirement to increase the adequacy of the instruments and methods used in the assessment of medication literacy. This does not mean excluding the metric and functional components of the assessment, but rather to emphasise the limitations of these assessments without a framework of other important social contextuality indicators.

From the focus on social contextuality discussed in this article, and empirically supported by the two studies presented, three components regarding the place of information in everyday literacy practices stand out as essential contributions to a socially contextualised conception of medication literacy. First, the composite nature of the informational resources mobilised by individuals in their relationship with medication, where expert information and practical knowledge co-inhabit laypersons’ reference models for their consumption; this informational pluralism invalidates the binary logic of informed/uninformed user that is prevalent in strictly technical and ethno-professional conceptions of literacy. Second, medication literacy, like other types of literacy, is socially constructed through *informational trajectories*; this notion allows us to capture the articulated ways in which users construct their knowledge about medicines, but also how, at different moments in their consumption trajectory, they seek different types of information and different sources. Third, the assimilation and validation of information are inseparable from the contexts of sociability and concrete contexts of action, where the sharing of practical and somatic knowledge gains social vitality in the logics of medication use.

Two other implications result from this conceptualisation of medication literacy. First, a methodological one, which points to the need to integrate literacy assessment studies into analytical models using mixed methods (both quantitative and qualitative techniques). This would allow the production of indicators of the information valued by users in their relationship with medication, as well as of the rationalities that validate their information options, based on qualitative and contextualised data. Second, at a conceptual and interdisciplinary level, the conceptualisation proposed in this article also implies the need to deepen theoretical work in this field using an intra- and interdisciplinary perspective, and stresses the need for an effective conceptual investment in the sociological component of medication literacy.

Mastering the sociological dimension of medication literacy is key to understanding the social rationalities and practices in this field, as a requirement for literacy promotion models that are effectively oriented towards a more layperson-centred approach.

## Notes

1. Project ‘Medicines and dietary supplements in performance consumptions: social practices, contexts and literacy’ (PTDC/SOCSOC/30734/2017) – financed by the Portuguese Foundation for Science and Technology (FCT). Conducted between 2018 and 2022, at the Centre for Research and Studies in Sociology (CIES) of the Iscte-University Institute of Lisbon, in partnership with the Center for Interdisciplinary Research Egas Moniz (CiiEM), of the Univerity Institute of Health Sciences Egas Moniz (IUEM), and Institute of Sociology of Oporto University (ISUP). The research was approved by the Ethics Committee of Egas Moniz (protocol code CE 857, February 2020).

2. Portugal follows the European Union regulation, which has been transposed into national law in the Medicinal Products Law (Decreto-Lei nº 176/2006, de 30 de Agosto).

3. Supplements are marketed in Europe as foods and the information that should be provided to consumers is set under specific regulation (Regulation (EU) no 1169/2011 of the European Parliament and of the Council of 25 october 2011). Essentially, the labelling of these products contains information on their composition, daily intake and safety warnings.

4. Project ‘Performance therapeutic consumptions among young people: trajectories and information networks’ (PTDC/CS-SOC/118073/2010) – financed by the Portuguese Foundation for Science and Technology (FCT). Conducted between 2012 and 2014 at the Centre for Research and Studies in Sociology (CIES) of the Iscte-University Institute of Lisbon, in partnership with the Center for Interdisciplinary Research Egas Moniz (CiiEM), of the University Institute of Health Sciences Egas Moniz (IUEM). The research was approved by the Ethics Committee of Egas Moniz (protocol code CE 180, November 2012).

5. Adapted from Goffman’s (1987) ‘total institution’ concept.

## References

[bibr1-13634593231211520] Barber N (1993) Drugs: From prescription only to pharmacy only. British Medical Journal 307(6905): 640. 8401046 10.1136/bmj.307.6905.640 PMC1678987

[bibr2-13634593231211520] Baszanger I (1998) La Migraine – connaissances descriptives, traitment et prevention, Rapport établi à la demande de la Mutuelle Génerale de l’Education Nationale, INSERM, pp.251–263.

[bibr3-13634593231211520] Bissell P Ward PR Noyce PR (2000) Appropriateness measurement: Application to advice-giving in community pharmacies. Social Science & Medicine (1967) 51(3): 343–359. 10.1016/s0277-9536(99)00458-x 10855922

[bibr4-13634593231211520] Bissell P Ward PR Noyce PR (2001) The dependent consumer: Reflections on accounts of the risks of non-prescription medicines. Health 5(1): 5–30.

[bibr5-13634593231211520] Brown P Calnan M (2012) Trusting on the Edge: Managing Uncertainty and Vulnerability in the Midst of Serious Mental Health Problems. Bristol: Policy Press.

[bibr6-13634593231211520] Chinn D (2011) Critical health literacy. A review and critical analysis. Social Science & Medicine (1967) 73(1): 60–67. 10.1016/j.socscimed.2011.04.004 21640456

[bibr7-13634593231211520] Clamote T (2010) Consumos terapêuticos e fontes de informação [Therapeutic consumption and sources of information]. In: Lopes N (ed.) Medicamentos e Pluralismo Terapêutico. Práticas e lógicas sociais em mudança [Medication and Therapeutic Pluralism: Changing Practices and Social Logics]. Porto: Afrontamento, pp.87–157.

[bibr8-13634593231211520] Clamote T (2015) Reverberações da medicalização: Paisagens e trajetórias informacionais em consumos de performance [Reverberations of medicalization: Infoscapes and informational trajectories in performance consumption]. Sociologia xxix: 35–57.

[bibr9-13634593231211520] Collins J (2000) Bernstein, Bourdieu and the New Literacy Studies. Linguistics and Education 11(1): 65–78.

[bibr10-13634593231211520] Cordina M Hämeen-Anttila K Lauri J , et al (2018) Health and medication literacy and the desire to participate in pharmacotherapy decision making - comparison of two countries. Research in Social and Administrative Pharmacy 14(9): 817–823. 29934278 10.1016/j.sapharm.2018.06.009

[bibr11-13634593231211520] Dixon-Woods M (2001) Writing wrongs? An analysis of published discourses about the use of patient information leaflets. Social Science & Medicine (1967) 52: 1417–1432. 10.1016/s0277-9536(00)00247-1 11286365

[bibr12-13634593231211520] European Comission (2009) Guideline on the Readability of the Labelling and Package Leaflet of Medicinal Products for Human Use. London: European Comission.

[bibr13-13634593231211520] Fox N Ward K , et al (2009) Pharma in bedroom. . .and kitchen. . .The pharmaceuticalisation of daily life. In: Williams S (ed.) Pharmaceuticals and Society-Critical Discourses and Debates, Wiley-Blackwell. pp.41–53.

[bibr14-13634593231211520] Freidson E (ed.) (1984) La Profession Médicale, 1st edn. Paris: Payot.

[bibr15-13634593231211520] Rodrigues CF Lopes N Hardon A (2019) Beyond health: Medicines, food supplements, energetics and the commodification of self-performance in Maputo. Sociology of Health & Illness 41(6): 1005–1022. 30847964 10.1111/1467-9566.12880 PMC6850569

[bibr16-13634593231211520] Gee JP (2015) The New Literacy Studies. In: Rowsell J Pahl K (eds) The Routledge Handbook of Literacy Studies. London and New York: Routledge. pp.35–48.

[bibr17-13634593231211520] Giddens A (1991) Modernity and Self-Identity: Self and Society in the Late Modern Age. Cambridge: Polity Press.

[bibr18-13634593231211520] Giddens A (1992) As Consequências Da Modernidade. Oeiras: Celta.

[bibr19-13634593231211520] Grime J Blenkinsopp A Raynor DK , et al (2007) The role and value of written information for patients about individual medicines: A systematic review. Health Expectations 10: 286–298. 17678517 10.1111/j.1369-7625.2007.00454.x PMC5060401

[bibr20-13634593231211520] Herber OR Gies V Schwappach D , et al (2014) Patient information leaflets: Informing or frightening? A focus group study exploring patients’ emotional reactions and subsequent behavior towards package leaflets of commonly prescribed medications in family practices. BMC Family Practice 15(1): 163–168. 25277783 10.1186/1471-2296-15-163 PMC4287479

[bibr21-13634593231211520] Lopes N (2009) Changing self-medication practices, lay knowledge and rationales. RCCS Annual Review 1: 36–54.

[bibr22-13634593231211520] Lopes N (2010) Consumos terapêuticos e pluralismo terapêutico [Therapeutic consumption and therapeutic pluralism. In: Lopes N (ed.) Medicamentos e Pluralismo Terapêutico. Práticas e Lógicas Sociais em Mudança [Medication and Therapeutic Pluralism: Changing Practices and Social Logics]. Porto: Afrontamento, pp.19–85, (org.).

[bibr23-13634593231211520] Lopes N Clamote T Raposo H , et al (2015) Medications, youth therapeutic cultures and performance consumptions: A sociological approach. Health 19(4): 430–448. 25331645 10.1177/1363459314554317

[bibr24-13634593231211520] Lopes N Pegado E Zózimo JR (2017) Ageing and memory medication: Social rationales and consumption practices. Sociology of Health & Illness 39(7): 1273–1287. 28597495 10.1111/1467-9566.12586

[bibr25-13634593231211520] Monaghan L (1999) Challenging medicine? Bodybuilding, drugs and risk. Sociology of Health & Illness 21(6): 707–734.

[bibr26-13634593231211520] Nathan JP Zerilli T Cicero LA , et al (2007) Patients’ use and perception of medication information leaflets. The Annals of Pharmacotherapy 41: 777–782. 17405820 10.1345/aph.1H686

[bibr27-13634593231211520] Nutbeam D (2008) The evolving concept of health literacy. Social Science & Medicine (1967) 67(12): 2072–2078. 10.1016/j.socscimed.2008.09.050 18952344

[bibr28-13634593231211520] Pantuzza LLN Do Nascimento E Botelho SF , et al (2021) Mapping the construct and measurement of medication literacy: A scoping review. British Journal of Clinical Pharmacology 87(3): 754–775.

[bibr29-13634593231211520] Papen U (2009) Literacy, learning and Health – A social practices view of health literacy. Literacy and Numeracy Studies 16(2): 19–34.

[bibr30-13634593231211520] Peerson A Saunders M (2009) Health literacy revisited: What do we mean and why does it matter? Health Promotion International 24(3): 285–396. 19372101 10.1093/heapro/dap014

[bibr31-13634593231211520] Pegado E Lopes N Zózimo J (2018) Pharmaceuticalisation and the social management of sleep in old age. Ageing and Society 38(8): 1645–1666.

[bibr32-13634593231211520] Pires C Vigário M Cavaco A (2015) Legibilidade das bulas dos medicamentos: revisão sistemática. Revista de Saúde Pública 49(4): 1–13. 10.1590/S0034-8910.2015049005559 PMC4386563 25741660

[bibr33-13634593231211520] Pouliot A Vaillancourt R Stacey D , et al (2018) Defining and identifying concepts of medication literacy: An international perspective. Research in Social and Administrative Pharmacy 14(9): 797–804. 29191647 10.1016/j.sapharm.2017.11.005

[bibr34-13634593231211520] Raposo H (2010) Consumos terapêuticos, percepção e gestão do risco [Therapeutic consumption, risk perception and management]. In: Lopes N (ed.) Medicamentos e Pluralismo Terapêutico. Práticas e Lógicas Sociais Em Mudança [Medication and Therapeutic Pluralism: Changing Practices and Social Logics]. Porto: Afrontamento, pp.159–222, (org.).

[bibr35-13634593231211520] Raposo H (2016) O risco e os consumos de performance na população jovem: Entre as conceções e as práticas. Revista Portuguesa de Saúde Pública 34(2): 186–195.

[bibr36-13634593231211520] Raposo H Rodrigues CF (2021) Super Humanos. In: Barbosa Pussetti (eds) Imperativos e investimentos de performance em contextos juvenis: perceções e formas de gestão do risco e da eficácia [Performance Imperatives and Investements in Youth Contexts: Perceptions and Ways of Managing Risk and Efficacy]. Lisbon: Colibri, pp.77–104.

[bibr37-13634593231211520] Raynor DK (2009) Addressing medication literacy: A pharmacy practice priority. International Journal of Pharmacy Practice 17(5): 257–259. 20214266

[bibr38-13634593231211520] Raynor DK Silcock J Knapp P , et al (2007) How do patients use medicine information leaflets in the UK? International Journal of Pharmacy Practice 15(3): 209–218.

[bibr39-13634593231211520] Rodrigues CF (2016) Medicines and therapeutic pluralism in Maputo: exploring modalities of trust and the (un)certainties of everyday users. Health, Risk & Society 18(7-8): 385–406.

[bibr40-13634593231211520] Rodrigues CF (2020) Self-medication with antibiotics in Maputo, Mozambique: Practices, rationales and relationships. Palgrave Communications 6(1).

[bibr41-13634593231211520] Samerski S (2019) Health literacy as a social practice: Social and empirical dimensions of knowledge on health and healthcare. Social Science & Medicine (1967) 226: 1–8. 10.1016/j.socscimed.2019.02.024 30831555

[bibr42-13634593231211520] Sauceda JA Loya AM Sias JJ , et al (2012) Medication literacy in Spanish and English: Psychometric evaluation of a new assessment tool. The Journal of the American Pharmaceutical Association (1912) 52(6): e231–e240. 10.1331/JAPhA.2012.11264 23229985

[bibr43-13634593231211520] WHO (1988) Self-Medication in Europe. Copenhagen: Regional Office for Europe.

[bibr44-13634593231211520] Williams SJ Seale C Boden S , et al (2008) Waking up to sleepiness: Modafinil, the media and the pharmaceuticalisation of everyday/night life. Sociology of Health & Illness 30(6): 839–855. 18444954 10.1111/j.1467-9566.2008.01084.x

